# Crovalimab: a new era in paroxysmal nocturnal hemoglobinuria management

**DOI:** 10.1097/MS9.0000000000002775

**Published:** 2025-01-30

**Authors:** Aiman Waheed, Muhammad Hamza Gul, Abdul Baseer Wardak, Muhammad Usama Bin Shabbir, Sabahat Touseef, Fatima Zafar, Helai Hussaini

**Affiliations:** a Rawalpindi Medical College, Rawalpindi, Pakistan; b Hayatabad Medical Complex, Peshawar, Pakistan; c Razia Bahlol Hospital, Kabul, Afghanistan; d Pakistan Institute of Medical Sciences, Rawalpindi, Pakistan; e Shifa College of Medicine, Rawalpindi, Pakistan; f Anaheim Regional Medical Center California, California, USA

**Keywords:** anti-c5 antibody, commodore trials, complement-mediated destruction, crovalimab, glycosylphosphatidylinositol, hemolysis, paroxysmal nocturnal hemoglobinuria, PIGA gene, PNH, terminal complement activation, transfusion reduction

## Abstract

Crovalimab, a new promising drug for the treatment of paroxysmal nocturnal hemoglobinuria (PNH) has emerged. This marks a significant advancement in the treatment of PNH and potentially other complement-mediated disorders. PNH is characterized by complement-mediated hemolysis, thrombosis, and bone marrow failure, leading to severe morbidity and mortality in affected individuals. PNH arises from a somatic mutation in the PIGA gene, leading to the loss of glycosylphosphatidylinositol (GPI)-anchored proteins on blood cells, making them vulnerable to complement-mediated destruction. Crovalimab is a new anti-C5 recycling antibody that offers a promising treatment by being administered subcutaneously every 4 weeks at a low volume. Clinical trials such as COMMODORE 3 have shown crovalimab’s effectiveness and high tolerability in PNH patients who have not previously used a C5 inhibitor. Crovalimab has been proven effective in maintaining hemoglobin levels, reducing the need for transfusions, and improving patient outcomes by inhibiting terminal complement activation.

Crovalimab, a new promising drug for the treatment of Paroxysmal nocturnal hemoglobinuria (PNH) has emerged. This marks a significant advancement in the treatment of PNH and potentially other complement-mediated disorders. PNH is a rare acquired hematologic illness, which is characterized by complement-mediated hemolysis, thrombosis, and bone marrow failure, leading to severe morbidity and mortality in affected individuals. The incidence of PNH has been rising, with studies suggesting an increase in diagnosis due to improved awareness and advances in detection methods, estimated at 1 to 5 cases per million people per year^[1]^. A somatic mutation in the PIGA gene results in the development of PNH, which eliminates glycosylphosphatidylinositol (GPI)-anchored proteins on blood cells, making them vulnerable to complement-mediated destruction. Current therapies for PNH include monoclonal antibodies that target complement component C5, such as ravulizumab and eculizumab^[2]^. Despite their efficacy, challenges remain, including the frequency of dosing and the need for intravenous administration.

A new monoclonal antibody called Crovalimab has shown promise in treating (PNH). Crovalimab was approved by the United States Food and Drug Administration (FDA) on 25 December 2022, based on clinical trial data demonstrating its efficacy in reducing hemolysis and improving patient outcomes, providing a subcutaneous dosing option that enhances patient convenience and adherence^[3]^. Its introduction marks a significant advancement in the management of PNH, reflecting ongoing efforts to improve patient care and quality of life in this rare hematologic disorder. The innate immune system’s complement cascade is crucial for eliminating damaged cells and thwarting infections.

Dysregulated complement mediated cascade activation in PNH is brought on by the lack of glycosylphosphatidylinositol (GPI)-anchored proteins on blood cells, leading to persistent hemolysis and thrombosis^[3]^. By binding specifically to C5, crovalimab inhibits its ability to cleave it into C5a and C5b, preventing the formation of the membrane attack complex (MAC) and complement-mediated hemolysis and thrombosis downstream^[3,4]^. Eculizumab and ravulizumab also target C5 but do so at distinct points in the complement cascade. By binding to C5, eculizumab prevents C5 convertase from cleaving it, stopping C5 from becoming C5a and C5b, which ultimately halts MAC production and complement-mediated cell lysis^[1,3]^. The next-generation complement inhibitor ravulizumab has a longer half-life than eculizumab, allowing for less frequent dosing with comparable efficacy in PNH treatment. Although all three therapies (ravulizumab, eculizumab, and crovalimab) inhibit complement-mediated hemolysis and thrombosis in PNH through targeting C5, each treatment offers unique benefits in terms of dosing frequency, patient convenience, and possibly improved efficacy in treating this complex hematologic disorder^[1,3,4]^.

To determine crovalimab’s effectiveness and safety in treating individuals with (PNH), multiple clinical trials have been conducted. The purpose of the COMPOSER trial was to evaluate crovalimab’s safety, pharmacokinetics, pharmacodynamics, and exploratory effectiveness. It included patients with PNH who had never taken a C5 inhibitor before, as well as patients with PNH who had switched from another C5 inhibitor. Results revealed that both naive and pretreated patients had prolonged suppression of the terminal complement pathway. Patients who had not received any treatment showed rapid and long-lasting decreases in hemolysis, lower rates of transfusions, and higher hemoglobin levels^[5]^. Participants in the COMMODORE 2 trial, which evaluated the safety and efficacy of eculizumab versus crovalimab, were PNH patients 12 years and older who had never received complement inhibitor therapy. This multicenter, randomized, open-label, active-controlled trial had a 2:1 dose ratio of eculizumab to crovalimab. Reduced hemolysis, elimination of transfusion requirements, and increased safety were the main objectives^[6]^. Crovalimab exhibits a tolerable safety profile along with prolonged complement inhibition, and its longer half-life is convenient for patients. A summary of the key findings of the trials mentioned is given in Table 1.

**Table 1:** Clinical trials of crovalimab in PNH: key findings and endpoints.

Trial Name	Year	Study Type	Key Findings	Primary Endpoints	Outcome Measures
**COMPOSER Study**^[5]^	2022	Phase 3, Open-label, Randomized Trial	Sustained reduction in hemolysis, increased hemoglobin levels, and transfusion independence in PNH patients.	Reduction in hemolysis, transfusion independence	LDH levels reduced by 87% (*P* < 0.001); 74% of patients achieved transfusion independence
**COMMODORE 2 Study**^[6]^	2023	Phase 3, Randomized Non-Inferiority Study	Comparable efficacy and safety of crovalimab vs. eculizumab; assessed reduction in hemolysis and transfusion independence.	Reduction in hemolysis, transfusion independence	LDH levels reduced by 85% with crovalimab vs. 83% with eculizumab (*P* = 0.002 for non-inferiority); similar rates of adverse events

This new anti-C5 recycling antibody, crovalimab, can be administered subcutaneously every 4 weeks at a low volume, which helps treat (PNH). Empirical research has shown the effectiveness and high tolerability of crovalimab in PNH patients who have not taken a C5 inhibitor. A similar safety profile to eculizumab, another well-researched medication for PNH, is indicated by pooled safety data from studies such as COMMODORE 1, 2, and 3. Comparable rates of adverse events have been documented for both drugs; the most frequent adverse event linked to crovalimab is infusion-related reactions^[5,7]^. The Phase III COMMODORE 1 trial evaluated the effectiveness and safety of crovalimab compared to eculizumab in patients with paroxysmal nocturnal hemoglobinuria who had previously utilized complement inhibitors. Essential outcomes in PNH care, such as hemoglobin stabilization and transfusion avoidance, showed that crovalimab and eculizumab were non-inferior. Additionally, the subcutaneous dose of crovalimab every 4 weeks was well-tolerated and provided a practical substitute for the biweekly intravenous delivery of eculizumab. Most reported adverse effects were mild to moderate, with the most common reactions occurring during infusion including headache, nausea, and infusion-related reactions such as chills and fever. These results show that crovalimab is a good therapy option for PNH patients who have previously used complement inhibitors, which may enhance patient satisfaction and treatment compliance^[8]^.

Healthcare decision-makers must assess the cost-effectiveness of crovalimab in treating (PNH). Compared to more established treatments like eculizumab, crovalimab may initially be more expensive. However, because of its less frequent dosage schedule and capacity to lower hospital stays, it may be more cost-effective in the long run, particularly in developed regions such as North America, Europe, and parts of Asia where healthcare systems are increasingly focused on optimizing expenditures. Countries such as the United States, Germany, and Japan can afford such innovative therapies, given their established healthcare funding mechanisms and willingness to invest in advanced treatment. Economic assessments consider variables such as treatment compliance, the use of healthcare resources, and indirect expenses related to illness management. Crovalimab offers a viable, cost-effective solution that aligns with healthcare cost-containment initiatives by enhancing patient outcomes and lowering complications. With continued research examining its potential in combination therapy and wider uses beyond complement-mediated diseases, crovalimab’s future in the treatment landscape goes beyond PNH. To maximize treatment outcomes and reduce disease burden, ongoing studies are assessing the safety and efficacy of crovalimab when used in conjunction with other complement inhibitors. Atypical hemolytic uremic syndrome and other uncommon disorders marked by complement dysregulation are among the conditions for which planned clinical trials intend to investigate the therapeutic potential of crovalimab^[9]^. These initiatives highlight how crovalimab is being used in personalized medicine and how it can help patients with complicated complement-mediated illnesses have more treatment options.

The treatment of PNH and possibly other complement-mediated illnesses has significantly advanced with crovalimab. Compared to traditional therapies, crovalimab has proven effective in maintaining hemoglobin levels, lowering the need for transfusions, and improving patient outcomes by suppressing terminal complement activation. Clinical trials such as COMMODORE have demonstrated the safety profile and tolerability of crovalimab, establishing it as a feasible substitute or supplement to currently available treatments such as eculizumab, ravulizumab. Plans are underway to investigate combination treatments with additional complement inhibitors and to broaden its use in uncommon illnesses marked by complement dysregulation. In short, crovalimab represents a promising therapeutic alternative to patients with complex hematologic disorders.

## Data Availability

Not applicable.

## References

[1] Brodsky RA. Paroxysmal nocturnal hemoglobinuria. Blood 2014;124:2804–11. 25237200 10.1182/blood-2014-02-522128 PMC4215311

[2] Risitano AM. Paroxysmal nocturnal hemoglobinuria and other complement-mediated hematological disorders. Immunobiology 2012;217:1080–87. 22964233 10.1016/j.imbio.2012.07.014

[3] Hillmen P Muus P Röth A. Long-term safety and efficacy of sustained eculizumab treatment in patients with paroxysmal nocturnal haemoglobinuria. Br J Haematol 2013;162:62–73. 23617322 10.1111/bjh.12347 PMC3744747

[4] Parker C Omine M Richards S. Diagnosis and management of paroxysmal nocturnal hemoglobinuria. Blood 2005;106:3699–709. 16051736 10.1182/blood-2005-04-1717 PMC1895106

[5] Röth A Nishimura J Nagy Z. The complement C5 inhibitor crovalimab in paroxysmal nocturnal hemoglobinuria. Blood 2020;135:912–20. 31978221 10.1182/blood.2019003399 PMC7082616

[6] Kulasekararaj A He G Munir T. Trial in progress: the phase III, randomized, open-label, multicenter COMMODORE 2 study evaluating the efficacy and safety of crovalimab versus eculizumab in adult and adolescent patients with paroxysmal nocturnal hemoglobinuria not previously treated with complement inhibitors. Blood 2020;136:34.

[7] PharmD BP Crovalimab under review for paroxysmal nocturnal hemoglobinuria. MPR. https://www.empr.com/home/news/drugs-in-the-pipeline/crovalimab-under-review-for-paroxysmal-nocturnal-hemoglobinuria/. Published September 8, 2023.

[8] Scheinberg P Cle D Edwards J. S183: PHASE III randomized, multicenter, open-label commodore 1 trial: comparison of crovalimab vs eculizumab in complement inhibitor-experienced patients with paroxysmal nocturnal hemoglobinuria (PNH). Hemasphere 2023;7:e45540d8. 10.1002/ajh.27413 38924124

[9] Samatharenigunta. PiaSky (crovalimab) for the treatment of Paroxysmal Nocturnal Haemoglobinuria (PNH), USA. Clinical Trials Arena. https://www.clinicaltrialsarena.com/projects/piasky-crovalimab-for-the-treatment-of-paroxysmal-nocturnal-haemoglobinuria-pnh/?cf-view. Published July 4, 2024

